# Dietary patterns in relation to incidence rate of pancreatic cancer – the Norwegian women and cancer cohort study

**DOI:** 10.29219/fnr.v67.9536

**Published:** 2023-09-29

**Authors:** Eliska Selinger, Charlotta Rylander, Guri Skeie

**Affiliations:** 1 Department of Epidemiology and Biostatistics, 3rd Faculty of Medicine, Charles University, Prague, Czech Republic; 2 Centre for Public Health Promotion, National Institute of Public Health, Prague, Czech Republic; 3 Department of Community Medicine, Faculty of Health Sciences, UiT The Arctic University of Norway, Tromsø, Norway

**Keywords:** Pancreatic cancer, dietary patterns, women, Norway, prospective cohort

## Abstract

Despite development in cancer treatment and prevention options during the past few years, cancer of the pancreas remains a diagnosis associated with poor prognosis and limited options for prevention. Diet has proven to be an important risk factor for development of many types of cancer, particularly for cancers of the digestive system. Still, evidence regarding its relation to pancreatic cancer remains ambiguous. To investigate the relationship between diet and pancreatic cancer, an analysis of dietary patterns in participants from the Norwegian Women and Cancer Study (*n* = 89,156; 305 pancreatic cancer cases) was performed. Cox regression analysis was used for studying possible associations between dietary patterns, derived from principal component analysis, and pancreatic cancer incidence. The four most prominent dietary patterns were identified and described: European pattern, animal food consumers’ dietary pattern, traditional Norwegian pattern, and alcohol-abstaining dietary pattern. In analysis without adjustment for confounders, being in the highest tertile of the abstaining dietary pattern was associated with lower risk of pancreatic cancer in comparison to the lowest tertile (hazard ratios [HR]: 0.66, 95% confidence interval [CI]: 0.49–0.89). After additional adjustment for height and smoking status, no dietary pattern was associated with increased pancreatic cancer risk, nor was there any difference in effect estimates between strata of smokers and non-smokers. The results of our current analysis do not support the role of major dietary patterns in the development of pancreatic cancer.

## Popular scientific summary

- Prevention of pancreatic cancer is among the priorities of cancer research
- Lifestyle factors are suspected to play a role in the risk of pancreatic cancer
- Several dietary factors are suspected to influence the risk with conflicting results
- We investigated the relation between whole dietary patterns (in contrast to specific foods) and risk of pancreatic cancer, and the results do not support a role of dietary patterns in the development of pancreatic cancer

Pancreatic cancer is one of the most lethal malignant cancers, responsible for almost half a million deaths per year globally and with a 5-year survival rate of less than 10% (1). Despite these numbers, there is still limited knowledge about different options for primary prevention. According to the World Cancer Research Fund (WCRF) (2), smoking is an established cause of pancreatic cancer. Besides that, there is strong evidence for body fatness being a cause of pancreatic cancer and probable evidence for adult height. However, only limited evidence currently exists for a relationship between specific dietary factors such as red and processed meat, foods containing saturated fatty acids, alcoholic drinks and foods and beverages containing fructose, and development of pancreatic cancer.

Research on nutritional factors involved in cancer development is complicated by several limitations. First, the relatively low incidence of many cancers and long latency period require large cohort studies with long follow-up to gain sufficient power to detect important differences. Moreover, the potential effect of nutrition is confounded by a range of non-dietary factors, like other lifestyle characteristics (e.g. physical activity) or socio-economic status as well as by intake of other dietary groups, challenging the interpretation of any findings. Single nutrients or single food usually have a limited impact on health outcomes. Moreover, when assessing overall health effects, it is essential to consider not only the specific food under investigation but also the potential effect of different substitutes if consumption changes. The interpretation of single-food studies is therefore limited as it may overlook the complexity of different dietary interactions existing in a real world. This has led to heated discussions about the reliability of such an approach, especially due to many discordances between findings of cohort studies and subsequent trials (3). In an attempt to at least partially compensate for these limitations associated especially with the single food approach, many advocate for a shift towards investigation of whole dietary patterns, which tend to bring more robust results across different types of studies, arguing to preserve single-nutrient analysis only as additional evidence (4–6).

Several methods of investigation of dietary patterns and their impact on health outcomes have been described (7, 8). Beside investigator-driven methods, using pre-specified dietary scores and pre-defined dietary patterns, data-driven methods have come to use. The basis for them is the utilization of exploratory statistical methods, like clustering methods, to identify dietary patterns naturally occurring in the population without any predetermined set of criteria. Characteristics related to the identified patterns can then be further investigated, including testing of their association with disease occurrence. One of the exploratory methods used for the identification of such habitual dietary patterns is the principal component analysis (PCA) (3, 9–11).

To investigate the potential relationship between diet and the incidence of pancreatic cancer, we decided to utilize data from a cohort of Norwegian women. The main aims of the present study are to identify dietary patterns adopted by the Norwegian female population using PCA and to analyze their relationship with the risk of pancreatic cancer.

## Methods

### Study design, setting, and available data

The Norwegian Women and Cancer (NOWAC) cohort study is a cohort recruited between the years 1991 and 2005. Women were randomly selected from the National registry and invited to participate in the study. A detailed profile of the cohort has been published elsewhere (12). Most of the recruited women completed a detailed validated food frequency questionnaire (FFQ) and self-reported important health information, for example, height, weight, use of hormonal contraceptives, hormonal replacement therapy, smoking, as well as socio-economic data like educational level and number of children (13–15). The FFQ used for recording of dietary information was validated in several methodological studies, including a biomarker study, a comparison with repeated 24 h dietary recalls, and used in a reproducibility study. When compared to 24 h dietary recalls, it was concluded that the FFQ demonstrated a commendable capacity to rank participants concerning frequently consumed foods (14–16). The data were linked with the Cancer Registry of Norway. The NOWAC study is conducted by a team of researchers mainly at UiT the Arctic University of Norway. This study was performed in line with the principles of the Declaration of Helsinki. Approval was granted by the Regional Committee for Medical Research Ethics in Northern Norway (P REK NORD 141/2008) and the Norwegian Data Inspectorate. Informed consent was obtained from all participants. The study results are reported in line with STROBE-nut guideline (17).

### Participants

**Fig. 1:**
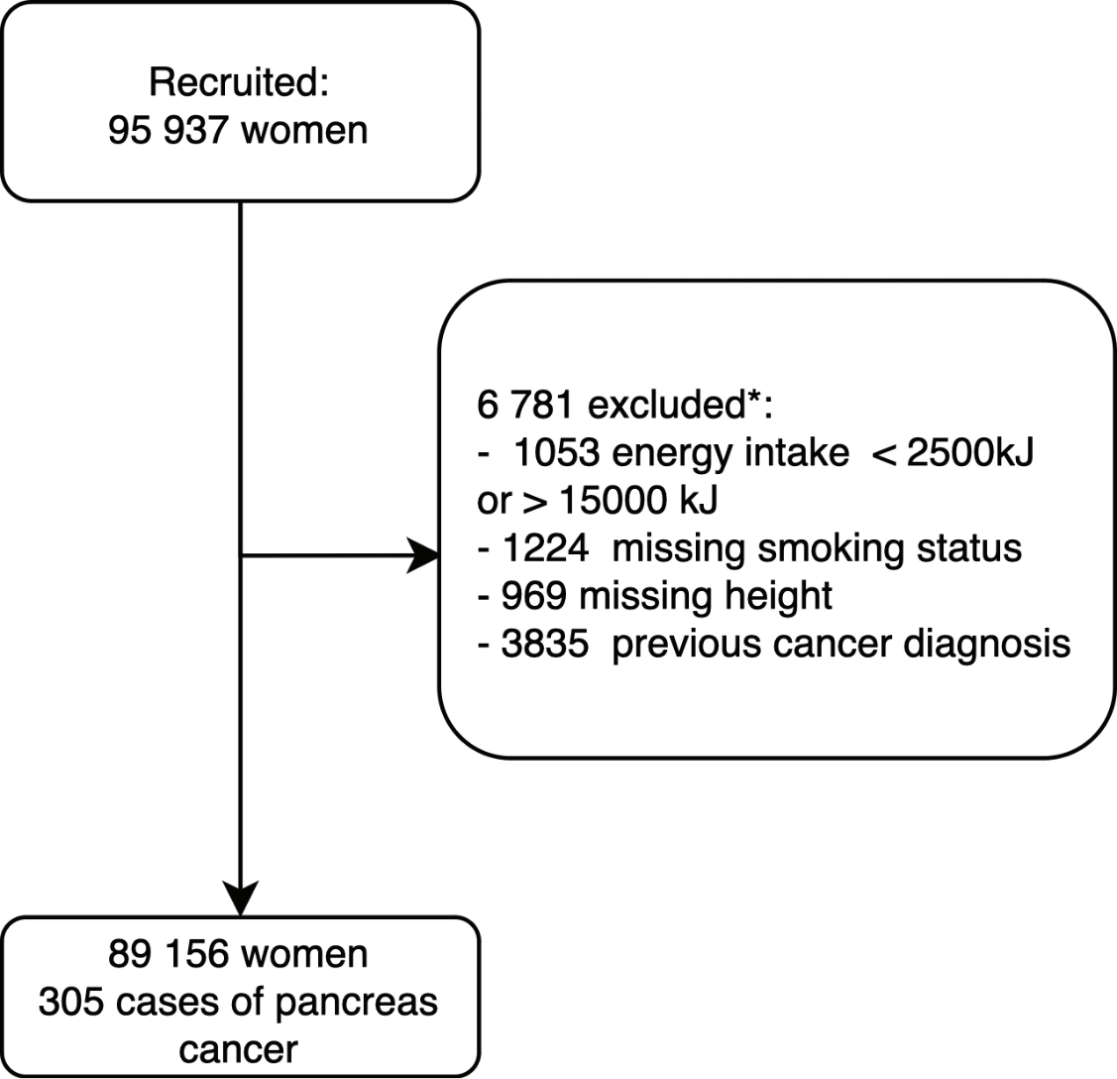
Flowchart of the selection process. Participants with unrealistic energy intakes or missing information on confounders/effect modifiers were excluded from the analysis. *Some participants had missing values for several variables, so the total number of excluded participants (6,781) is lower than the sum of the missing values.

A sample of 95,937 women was selected from study participants recruited in several waves between 1995 and 2005. Women reporting extreme energy intake in the food-frequency questionnaire (under 2,500 kJ or over 15,000 kJ) were excluded to decrease the risk of unrealistic dietary reporting distorting the results of the dietary patterns analysis (*n* = 1,053). Moreover, due to the planned survival analysis, women with missing information on smoking status (*n* = 1,224), height (*n* = 969), or women with previous cancer diagnoses were excluded before the analysis of dietary patterns (*n* = 3,835), with some of the participants missing information on more than one of these variables. Therefore, altogether, 6,781 persons were excluded from the analyses. The resulting sample consisted of 89,156 cancer-free women. In the final sample, 305 new cases of pancreatic cancer were recorded during the follow-up period. Details of the selection process are presented in Fig. 1.

### Principal component analysis

For the PCA, 73 to 90 original variables were retrieved from the FFQ and pre-grouped into 39 food variables (see Supplementary Table 1 for the detailed list of specific foods included in the grouped food variables). It is known that these decisions play a crucial role in the estimations of disease-exposure relationship, with research indicating that more detailed information on food groups intake results in enhanced precision of disease risk assessments (18). Due to this, decision on grouping was based not only on the nutritional value of a given food, but also on its known association with health and usage in Norwegian cuisine. Variables containing data on sweet beverages and nuts consumption were excluded from the analysis, as not all participants were asked about their intake in the FFQ used in their wave of recruitment. After the re-grouping and exclusion, Bartlett test of sphericity and Kaiser-Mayer-Olkin tests were performed to confirm the suitability of data for the analysis. The Bartlett’s test of sphericity was significant at an alpha level of 0.05 (*P* < 0.001), with overall Keiser-Meyer-Olkin value of 0.74. PCA was then applied to identify dietary patterns present in the obtained data. The food group intake values were standardized to reduce the influence of food intakes with high variance in the PCA and varimax rotation was performed on the loadings of PCA. Visual inspection of eigenvalues and percentage of variance explained were used as a measure for identification of the most prominent principal components (PCs) and their main dietary components were described in detail (main variables are defined as the 15 variables with the highest absolute value of eigenvalues). Full scree plots obtained from PCA analysis, including scree plots of variable loadings for selected components, can be found in Supplementary Figs. 1 and 2. Individual scores for selected PCs were retrieved (Supplementary Table 2), divided into tertiles, and associated background characteristics were assessed: age (in years), body mass index (BMI) (in kg/m^2^ and divided into two categories: <25 kg/m^2^ and BMI ≥ 25 kg/m^2^), height (in cm), number of children (categorized as no children, 1–2 children, more than two children), education level (years of schooling, categorized as 0–9 years, 10–12 years, more than 12 years) and smoking status (current smoker, previous smoker, non-smoker). The characteristics were described as mean and standard deviations or proportions, in case of categorical variables. All work was done using R software version 4.2.2, the FactoMineR (19) and factoextra (20) packages.

**Fig. 2:**
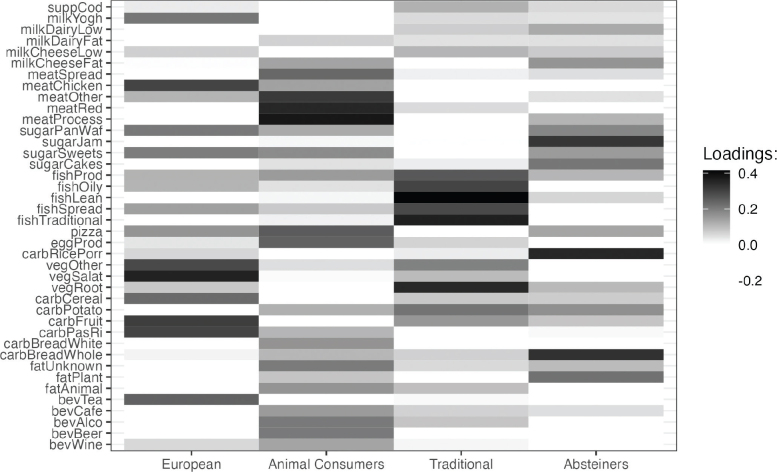
Principal component analysis: Main components loadings for four most prominent dietary patterns identified.

### Survival analysis

Cox regression models were built to describe the relationships between the identified dietary patterns, used as an independent exposure, and incidence of pancreatic cancer as outcome. Incident cases of pancreatic cancer were defined as those given code C25 from the International Classification of Diseases version 10 (ICD-10). For the purpose of the survival analysis, attained age in years was used as time scale. The end of follow-up was defined as the age at the end of the study period (31.12.2016), age at emigration, age at death, or cancer diagnosis, whichever came first.

Three models were constructed, one with mutual adjustment for identified dietary patterns, and two multivariable-adjusted models, one adjusting for height and smoking and another one with additional adjustment for BMI. Based on previous research describing their strong relation to the risk of pancreatic cancer, smoking status (non-smokers, ex-smokers, smokers) and height were used as additional independent variables in the multivariable model 2. Moreover, analyses stratified on smoking status were performed due to the possibility of effect modification of the dietary risk. Smoking status was divided into two strata based on baseline reported smoking status (current non-smokers, current smokers). BMI and energy intake were considered to be mediators of the dietary effect and therefore was not included in the main models. However, it is important to take into account also the possibility that the relationship between diet and BMI can be more complex. Diet itself can influence the body weight, but BMI can also influence the reported dietary intake. Also, there was a relationship with dietary intake as well as tendency for the association with the outcome of interest in our dataset (mean BMI in cases = 25.2 vs. non-cases 24.7; *P* = 0.05). Therefore, an additional model with BMI added as a confounder was constructed to explore the direct association between any of the dietary patterns not mediated through the effect of body weight. Results of the analyses are reported as hazard ratios (HR), together with corresponding 95% confidence intervals (CI). Statistical analysis was undertaken using R software version 4.2.2, Survival package (21).

## Results

### Characteristics of the sample

After the exclusions, 89,156 women were included in the analyses contributing with 1,424,465.9 person-years of follow-up. The median follow-up time of participants was 15.9 years (min: 0.03 years, max: 20.58 years). Mean age at the time of enrolment was 51.6 years. Detailed characteristics of the final sample are presented in Table 1.

**Table 1:** Baseline characteristics of the study sample (*n* = 89,156)*

Baseline characteristics	Mean	SD
Age at enrolment (years)	51.6	6.3
Height (cm)	166.2	5.7
Weight (kg)	68.3	11.6
BMI (kg/m^2^)	24.7	4.0
	***n***	**Percentage, %**
**Smoking status**		
Non-smoker	33,227	35
Previous smoker	32,590	35
Smoker	27,924	30
**BMI category**		
BMI < 25 kg/m^2^	55,475	60
BMI ≥ 25 kg/m^2^	36,367	40
**Number of years in school**		
0–9 years	23,133	26
10–12 years	30,306	34
More than 12 years	35,204	40
**Number of children**		
No children	8,100	9
1–2 children	49,067	52
More than two children	36,574	39

* Total number of participants may differ between the variables due to various missing values.

### Dietary patterns

After the PCA was performed and scree plot examined, four main dietary patterns were identified: European pattern, animal food consumers’ dietary pattern, traditional Norwegian pattern, and alcohol-abstaining dietary pattern. A detailed description of the loadings of each variable associated with the identified dietary patterns after varimax rotation can be found in Fig. 2, Supplementary Table 3 and Supplementary Fig. 3. Description of relevant background characteristics of participants per pattern is presented in Supplementary Table 2.

#### PC1: European diet

This dietary pattern was characterized by high consumption of vegetables (particularly salad vegetables and other vegetables), fruit, pasta, or rice, but also pizzas, waffles, and sweets. As protein, chicken, and yoghurt were prominent. Potatoes and fish spreads were consumed in lower amounts than in other patterns. Participants with high scores for this pattern tended to be relatively younger, more educated, and not have children. Also, the highest tertile had a lower proportion of current smokers compared to the lowest tertile.

#### PC2: Animal food consumers

This dietary pattern was characterized by high consumption of meat, including processed and red meat and meat spreads, eggs, and animal fat. Reported drinking of hard liquors and beer is also apparent. Women in the highest tertile of the score for this pattern were younger compared to those in the lowest tertile, less educated, tended to have more children and were current smokers.

#### PC3: Traditional Norwegian diet

The traditional Norwegian dietary pattern was characterized by a high intake of all kinds of fish and fish products, including cod liver oil, fruit, and vegetables (especially root vegetables) and potatoes. At the same time, participants with high scores for this pattern tended to eat less salad vegetables, sweets, and pizzas. Women in the highest tertile for this pattern tended to be older on average, less educated and non-smokers compared to women in the lowest tertile.

#### PC4: Abstainers

The most prominent characteristic of the fourth dietary pattern was the low consumption of all kinds of alcohols (liquors, wine, beer), accompanied by higher consumption of wholemeal bread, porridge, cakes, jam, and sweets. Participants in the highest tertile were more educated, more likely to be non-smokers and a higher proportion of them had more than two children compared to women in the lowest tertile.

### Risk of pancreatic cancer

Being in the highest tertile for the abstaining dietary pattern was inversely associated with pancreatic cancer risk compared to the lowest tertile (HR 0.66, 95% CI: 0.49–0.89) in the model with mutual adjustment for all four dietary patterns. No clear relationship was apparent with the other dietary patterns. After adjustment for height and smoking status, we observed no substantial association between any of the dietary patterns and incidence of pancreatic cancer. Additionally, a model adjusted also for the BMI status (normal weight or overweight and obese) was prepared to investigate the direct association of dietary patterns on pancreatic cancer without confounding by body weight. There were no notable changes in the estimates after this additional adjustment. Detailed results for unadjusted and adjusted models are reported in Table 2.

**Table 2:** Association between dietary patterns and risk of pancreatic cancer in the Norwegian Women and Cancer study. Tertiles of individual scores for dietary patterns compared.

		*n*	*n* of cases	Model l	Model 2	Model 3
				HR (95% CI)	HR (95% CI)	HR (95% CI)
PC1: European	1st tertile	29,719	134	Ref.	Ref.	Ref.
	2nd tertile	29,718	103	1.35 (1.04 – 1.76)	1.42 (1.09 – 1.84)	1.43 (1.10 – 1.86)
	3rd tertile	29,719	64	0.83 (0.60 – 1.16)	0.92 (0.66 – 1.0)	0.92 (0.66 – 1.29)
PC2: Animal food consumers	1st tertile	29,719	112	Ref.	Ref.	Ref.
	2nd tertile	29,718	97	1.09 (0.83 – 1.44)	1.07 (0.81 – 1.41)	1.04 (0.79 – 1.38)
	3rd tertile	29,719	92	1.06 (0.80 – 1.41)	0.98 (0.74 – 1.31)	0.97 (0.73 – 1.29)
PC3: Traditional Norwegian	1st tertile	29,719	75	Ref.	Ref.	Ref.
	2nd tertile	29,718	92	0.96 (0.71 – 1.30)	0.99 (0.73 – 1.34)	0.95 (0.69 – 1.29)
	3rd tertile	29,719	134	0.97 (0.72 –1.32)	1.03 (0.77 – 1.40)	1.02 (0.76 – 1.38)
PC4: Abstainers	1st tertile	29,719	123	Ref.	Ref.	Ref.
	2nd tertile	29,718	109	0.87 (0.67 – 1.12)	1.02 (0.78 – 1.33)	1.03 (0.79 – 1.34)
	3rd tertile	29,719	69	0.66 (0.49 – 0.89)	0.86 (0.63 – 1.16)	0.85 (0.62 – 1.16)

Model 1: Mutual adjustment of the four dietary patterns only.

Model 2: Model 1 + adjustment for height and smoking.

Model 3: Model 2 + BMI group.

### Stratification

While smoking status by itself was associated with the incidence of pancreatic cancer, no dietary pattern was associated with pancreatic cancer risk in strata of current smokers or non-smokers, adjusted for height. The full results of the stratified analyses are shown in Table 3.

**Table 3:** Association between dietary patterns and risk of pancreatic cancer in the Norwegian Women and Cancer study, stratified by smoking status. Tertiles of individual scores for dietary patterns compared, adjusted for height.

		Current smokers	Current non-smokers
Total *n* of participants:		26,717	62,439
Total *n* of events:		143	158
Multivariable model*		HR (95% CI)	HR (95% CI)
PC1: European	1st tertile	Ref.	Ref.
	2nd tertile	1.91 (1.32 – 2.76)	1.04 (0.71 – 1.52)
	3rd tertile	0.77 (0.44 – 1.32)	1.00 (0.65 – 1.53)
PC2: Animal food consumers	1st tertile	Ref.	Ref.
	2nd tertile	1.27 (0.83 – 1.94)	0.93 (0.65 – 1.35)
	3rd tertile	1.22 (0.80 – 1.86)	0.83 (0.56 – 1.23)
PC3: Traditional Norwegian	1st tertile	Ref.	Ref.
	2nd tertile	0.97 (0.64 – 1.46)	1.05 (0.66 – 1.67)
	3rd tertile	0.83 (0.54 – 1.28)	1.27 (0.82 – 1.96)
PC4: Abstainers	1st tertile	Ref.	Ref.
	2nd tertile	1.08 (0.75 – 1.58)	0.91 (0.63 – 1.32)
	3rd tertile	1.07 (0.68 – 1.67)	0.70 (0.47 – 1.05)

* Results from Cox regression, with mutually adjusted dietary patterns and adjustment for height.

## Discussion

We revealed four main dietary patterns among Norwegian women: a European pattern, characterized by high intake of fruit, vegetables, pasta and rice, lean meat but also sweets, an animal food consumers’ pattern with a high intake of all kinds of animal-based food, except for fish, a traditional Norwegian pattern based mainly on the consumption of fish and fish products together with potatoes and root vegetables, and an alcohol-abstaining dietary pattern, characterized by the low intake of all kinds of alcoholic drinks. Despite some evidence for a relation between the alcohol-abstaining dietary pattern and the risk of pancreatic cancer in the analysis with mutual adjustment for all four dietary patterns, no dietary pattern was found to have any apparent association with the risk of pancreatic cancer development after adjustment for known confounders.

As there is a possibility of effect modification by smoking status, or in other words diet having different associations with pancreatic cancer in smokers versus non-smokers, a stratified analysis was performed. No strong evidence for any relationship was found for any of the strata or dietary patterns. Neither did our estimates change much when the main model was additionally adjusted for BMI.

Several previous studies have attempted to describe the possible relation between food intake, diet, or lifestyle and pancreatic cancer. No relationship was found with intake of vegetables, fruit, or fiber (22). Another analysis of the European Prospective Investigation into Cancer and Nutrition (EPIC) cohort data observed a relationship between a healthy lifestyle index and pancreas cancer. High score on a healthy lifestyle index based on a combination of smoking, alcohol intake, dietary exposure, physical activity, and waist-to-hip was associated with a lower hazard of pancreas cancer. After the removal of smoking, Population attributable fraction (PAF) for pancreas cancer and lifestyle was estimated as 14% (95% CI: 6–21) (23). Moreover, two previously published systematic reviews and meta-analyses identified an inverse association of risk of pancreatic cancer and patterns with high intake of fiber, vitamins and vegetables or light-drinking of alcohol, while reporting positive associations between pancreatic cancer risk and Western dietary patterns (24, 25). Regarding the relationship between pancreatic cancer risk and dietary patterns, a recent umbrella review published in 2022 recognized an association between healthy plant-based diet and lower risk of pancreatic cancer. Conversely, high intake of red meat was associated with increased risk of pancreatic cancer. However, the general quality of evidence was low to critically low (26). Similar results were obtained in a multi case-control study published shortly after the umbrella review. Its aim was to evaluate the relationship between previously defined pro-vegetarian dietary patterns and risk of selected gastrointestinal cancers. In this study, comparing the relative risk ratios (RR) of the highest and the lowest quintiles for healthy pro-vegetarian pattern found a higher adherence to healthy pro-vegetarian pattern to be associated with a lower risk of pancreas cancer (RRR: 0.74, 95% CI: 0.59–0.92) (27). Those findings are in line with another meta-analysis concluding that plant-based diet is associated with lower risk of digestive system neoplasms, especially with pancreatic cancer (adjusted RR 0.71, 95% CI: 0.59–0.86) (28). In our study, we did not identify one specific pattern as plant-based, as different plant-based food groups loaded more strongly for different patterns, except the animal food consumption pattern.

Our results therefore differ from the previously reported studies as they do not confirm strong association between any identified dietary pattern and pancreatic cancer. Possible reasons for the differing conclusion can be different background risk in the populations involved as well as differences in dietary assessment methods or the methods used to identify the score for the dietary pattern with some studies using pre-specified criteria while others relying on posteriori methods, for example, cluster, factor or PCA. Differences in definitions and statistical methods used can lead to miscellaneous food groups combined in the patterns and can be the underlying reasons for the heterogeneous conclusions reported in available research. On the other hand, the most up-to-date report by WCRF still mentions only two confirmed causes of pancreatic cancer with strong evidence (having overweight or obesity, and being tall), and several other causes with some evidence (red meat, processed meat, smoking, and alcoholic drinks) (2). Our results add to the evidence of no clear association between dietary patterns including dietary patterns characterized by relatively high intakes of processed and red meat. In our results, there was tendency of lower risk associated for the dietary pattern with low alcohol consumption, but after adjustment for other confounders, even that relationship disappeared.

There are several limitations in our analysis. First, dietary data as well as other relevant information (smoking status and height) were self-reported by the participants. This creates potential for measurement bias which could lower our confidence in the obtained results. To limit this effect, only validated FFQs were used to collect the information, and participants with unrealistically reported intakes (defined as energy intakes under 2,500 kJ or over 15,000 kJ) were excluded from the analysis. Even though the number of cases in the total sample should be sufficient for the main analysis (*n* = 305), in the stratified analysis the number of cases per group was lower, reducing the overall power of the analysis. Therefore, the results of the stratified analyses should be interpreted with caution. To reduce confounding, multivariable models were constructed accounting for confounders selected based on available evidence. However, there could be other potential risk factors for pancreatic cancer that we could not adjust for, for example, family history. Another limitation is the inability to account for a family history or current diagnosis of diabetes, despite diabetes being a known risk factor for the development of pancreatic cancer (29). Due to this, residual confounding cannot be excluded.

## Conclusion

We observed no association between dietary patterns and risk of pancreatic cancer, nor was there any difference in associations between smokers and non-smokers. The results of our current analysis do not support a role of major dietary patterns in the development of pancreatic cancer.

## Conflict of interest and funding

The publication charges for this article have been funded by a grant from the publication fund of UiT the Arctic University of Norway, and the Healthy Choices project (ref. 289440). The work performed was funded by the Erasmus+ Programme. The authors declare no potential conflicts of interest.
