# Supplementary material for: Dietary patterns in relation to incidence rate of pancreatic cancer – the Norwegian women and cancer cohort study
Source: Food Nutr Res. 2023 Sep 29;67:10.29219/fnr.v67.9536. doi: 10.29219/fnr.v67.9536 (PMC10552709; doi:10.29219/fnr.v67.9536)
Supplement: Supplementary file 1 [file FNR-67-9536-s001.pdf]

Supplementary Figure 1: Eigenvalues for the identified principal components.

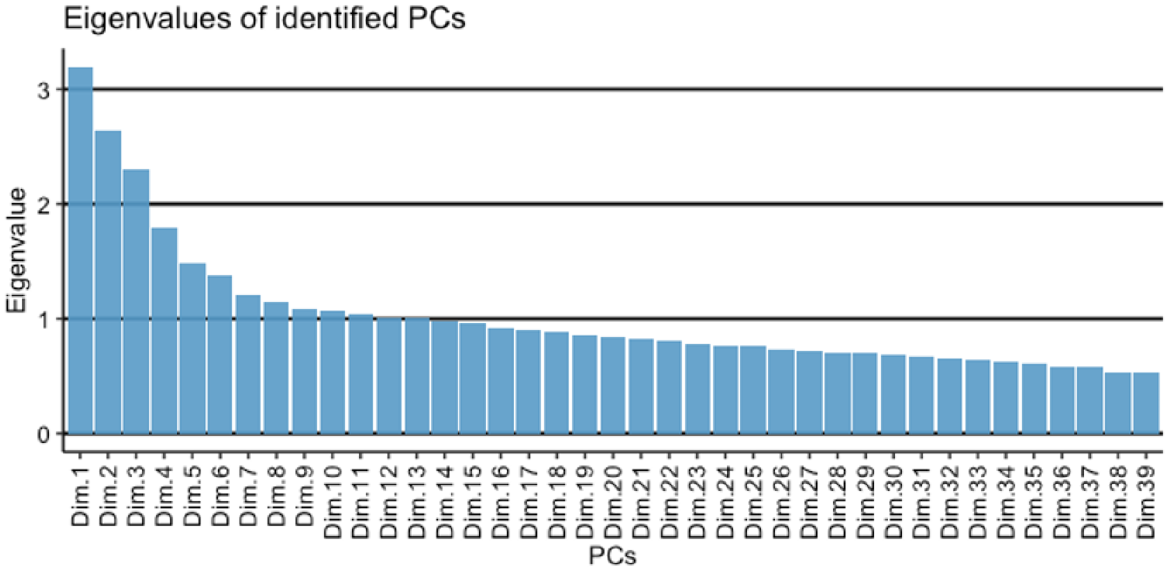

Supplementary Figure 2: Scree plot for the identified principal components.

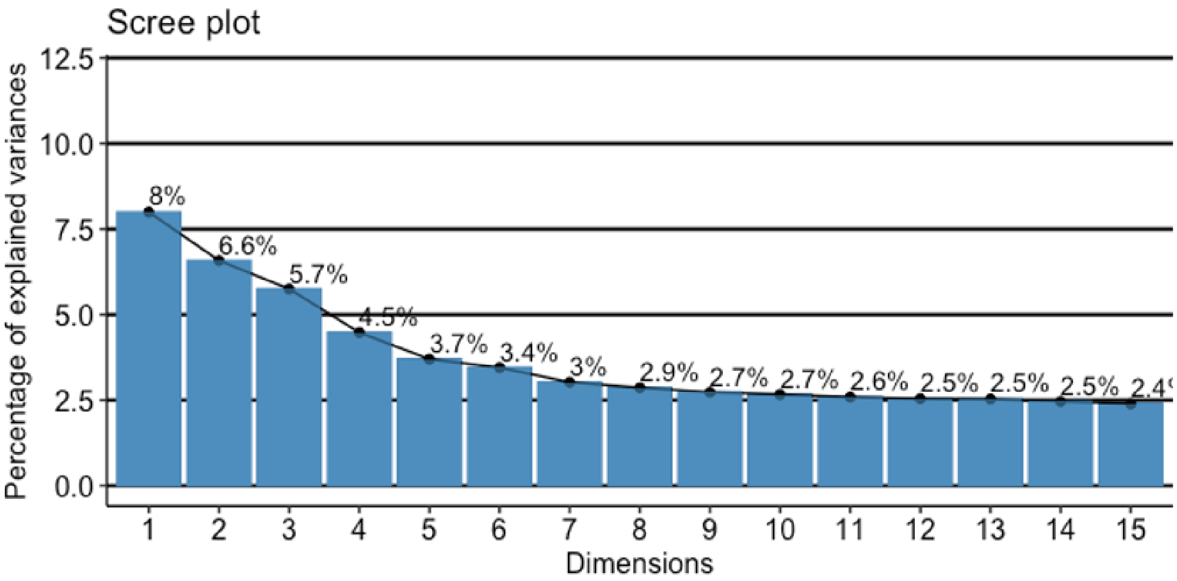

Supplementary Figure 3: Most prominent food variables for the main dietary patterns.

PC1: European diet

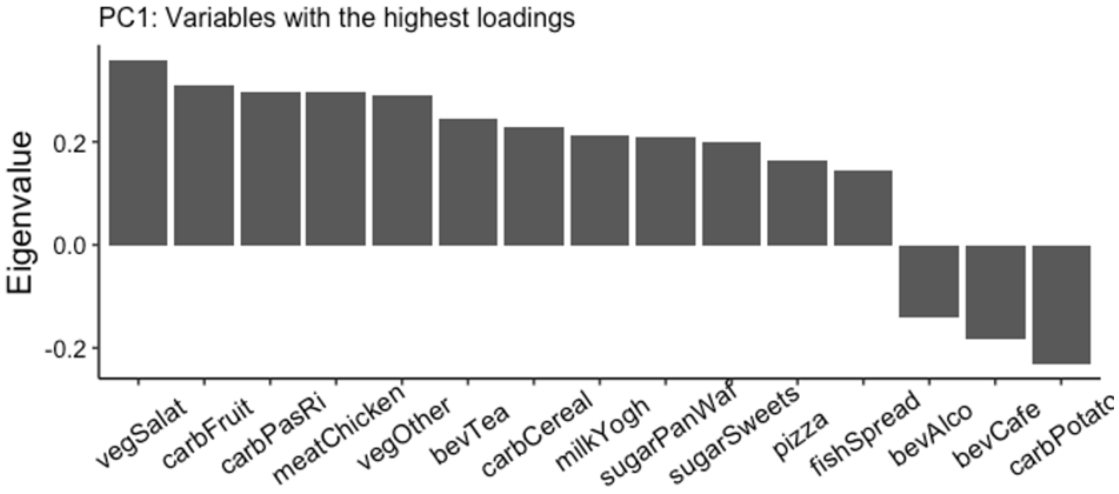

PC2:Animal food consumers

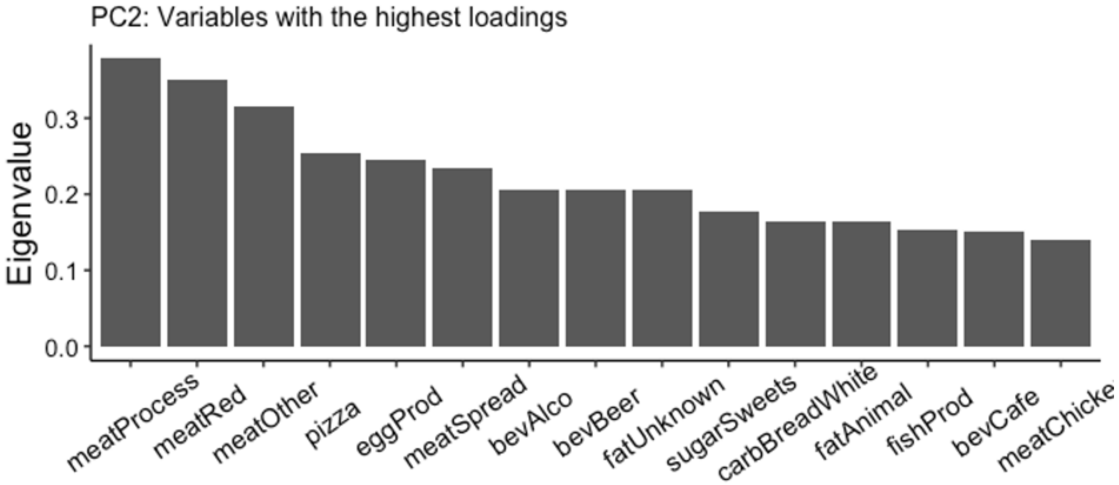

PC3: Traditional Norwegian diet

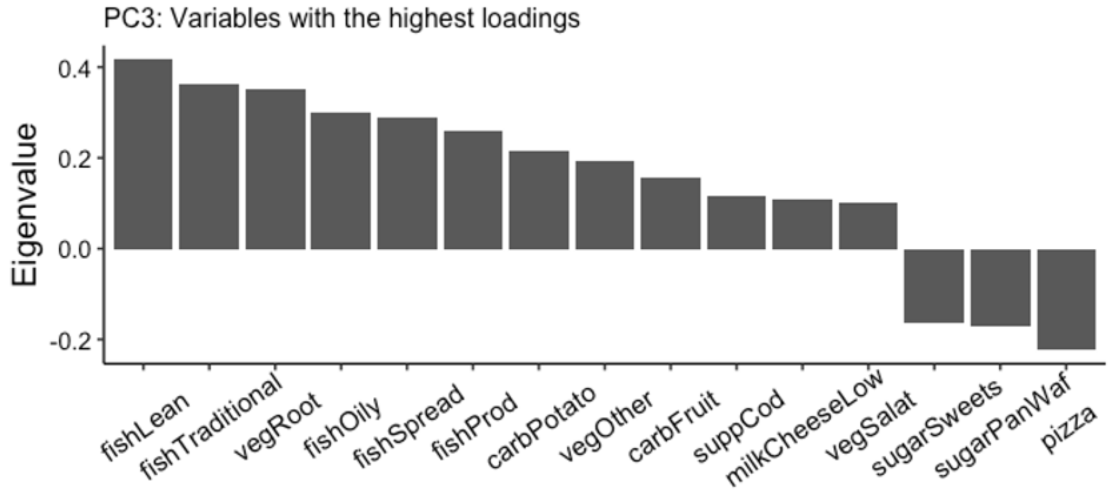

PC4: Abstainers

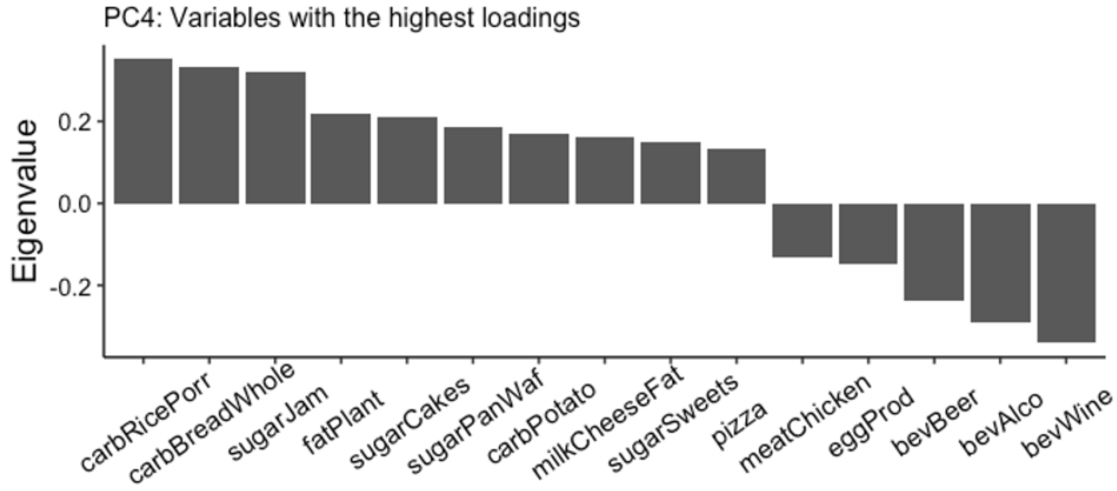

Supplementary Table 1: Detailed content of grouped variables used in principal component analysis.

Variable name	Food groups
**Alcohol – Wine**	Wine
**Alcohol – Beer**	Beer
**Alcohol - Liquor**	Liquor, spirits, or drinks based on liquor/spirit
**Beverages Coffee**	Coffee, boiled coffee, instant coffee, filter coffee, espresso
**Beverages Tea**	Green tea, black tea
**Carbohydrate – Wholemeal bread**	Whole grain bread types, crispbread
**Carbohydrate – White bread**	White bread, rolls
**Carbohydrate – Pasta and rice**	Pasta, rice
**Carbohydrate – Fruit**	Oranges, bananas, apples and pears and other kinds of fruit or fruit desserts
**Carbohydrate – Potatoes**	Potatoes
**Carbohydrate – Cereals**	Cereals and muesli
**Carbohydrate – Porridge**	Rice porridge, other kinds of porridge
**Carbohydrate - Vegetables, root**	Carrot, rutabaga, cabbage
**Carbohydrate - Vegetables, salad**	Salad, tomato
**Carbohydrate - Vegetables, other**	Broccoli and cauliflower and other kinds of vegetables
**Eggs**	Eggs
**Fat of animal origins**	Bacon fat/lard, butter, mayonnaise
**Fat of plant origin (by majority)**	Margarine, margarine and butter mixture of different fat contents and brands
**Fat of unknown origin**	Sauce containing fat
**Fish Lean and shellfish**	Cod (boiled, fried), general lean fish, wolffish and similar, shellfish
**Fish Oily**	Salmon, mackerel, tuna
**Fish spreads and fish eaten on bread**	Fish spreads, caviar spread, roe, salmon spread on bread, pickled herrings for bread, sardines for bread
**Fish products, composite dishes with fish**	Fish au gratin, fish cakes, fish soup, and deep-fried cod and similar
**Meat Processed**	Meatballs, sausage, added meat and meat products
**Meat Red**	Beef, chops, reindeer
**Meat Dishes with meat**	Stew and other kind of meat dishes
**Meat Chicken**	Chicken
**Meat spread**	Meat on bread
**Milk - Cheese, high fat**	Brown cheese (general + fatty variant), white cheese
**Milk - Cheese, low fat**	Brown cheese light variant, white cheese fat reduced variant

**Milk - High fat dairy**	Full cream milk, sour cream full fat, sour cream reduced fat
**Milk - Low fat dairy**	Skimmed milk, semi skimmed milk
**Milk – Yoghurt**	Yoghurt
**Pizza**	Pizza
**Sugar - Cakes, and baked goods**	Danish pastries, cakes, buns, cookies
**Sugar – Sweets**	Ice cream, chocolate, sugar in milk and coffee, desserts
**Sugar – Jam**	Jam
**Sugar – Pancakes and waffles**	Waffles and pancakes
**Supplement - Cod liver oil**	Cod liver oil

Supplementary Table 2. Baseline characteristics in tertiles of individual scores for the four main dietary patterns.

Variable	Dietary pattern		1st tertile	2nd tertile	3rd tertile	p-value
Age at baseline	PC1: European	Mean (SD)	53.4 (7.2)	50.5 (5.9)	50.7 (5.2)	< 0.001
	PC2: Animal food consumers	Mean (SD)	52.0 (6.3)	51.4 (6.3)	51.2 (6.2)	<0.001
	PC3: Traditional Norwegian	Mean (SD)	48.9 (5.2)	51.6 (6.0)	54.2 (6.5)	<0.001
	PC4: Abstainers	Mean (SD)	51.9 (6.1)	51.8 (6.5)	50.9 (6.2)	<0.001
Mean BMI	PC1: European	Mean (SD)	24.8 (4.0)	24.7 (3.9)	24.7 (3.9)	<0.001
	PC2: Animal food consumers	Mean (SD)	24.7 (3.9)	24.7 (3.9)	24.8 (4.1)	<0.001
	PC3: Traditional Norwegian	Mean (SD)	24.5 (4.0)	24.7 (3.9)	25.0 (4.0)	<0.001
	PC4: Abstainers	Mean (SD)	24.8 (4.0)	24.9 (4.0)	24.5 (3.8)	<0.001
Number of children N (column proportion)	PC1: European	No children	2430 (8.2)	2423 (8.2)	2777(9.3)	< 0001
		1-2 children	12900 (43.4)	16104 (54.2)	17720 (59.6)	
		More than 2 children	14389 (48.34	11191 (37.7)	9222 (31.0)	
	PC2: Animal food consumers	No children	3053 (10.3)	2420 (8.1)	2157 (7.2)	<0.001
		1-2 children	16460 (55.4)	15633 (52.6)	14631 (49.2)	
		More than 2 children	10320 (34.7)	11606 (39.1)	12876 (43.3)	
	PC3: Traditional Norwegian	No children	2285 (7.7)	2510 (8.4)	2835 (9.5)	<0.001
		1-2 children	16508 (55.5)	15778 (53.1)	14438 (48.6)	
		More than 2 children	10926 (36.8)	11430 (38.5)	12446 (41.9)	
	PC4: Abstainers	No children	2874 (9.7)	2379 (8.0)	2377 (8.0)	<0.001
		1-2 children	16525 (55.6)	15733 (52.9)	14466 (48.7)	
		More than 2 children	10373 (34.9)	11578 (39.0)	12851 (43.2)	
Education level N (column proportion)	PC1: European	0-9 years of school	12015 (43.9)	6421 (22.7)	3280 (11.4)	< 0.001
		10-12 years of school	8981 (32.8)	10594 (37.4)	9350 (32.5)	

		More than 12 years	6348 (23.2)	11300 (39.9)	16125 (56.1)	
	PC2: Animal food consumers	0-9 years of school	6712 (24.2)	7052 (24.9)	7952 (28.0)	<0.001
		10-12 years of school	9317 (33.6)	9690 (34.2)	9918 (34.9)	
		More than 12 years	11659 (42.1)	11550 (40.8)	10564 (37.2)	
	PC3: Traditional Norwegian	0-9 years of school	5878 (20.4)	7140 (25.3)	8698 (31.8)	<0.001
		10-12 years of school	10630 (36.9)	9752 (34.5)	8543 (31.3)	
		More than 12 years	12309 (42.7)	11371 (40.2)	10093 (36.9)	
	PC4: Abstainers	0-9 years of school	8075 (28.8)	7728 (27.6)	5913 (20.9)	<0.001
		10-12 years of school	10024 (35.7)	9676 (34.5)	9225 (32.6)	
		More than 12 years	9979 (35.5)	10636 (37.9)	13158 (46.5)	
Smoking status N (column proportion)	PC1: European	Non-smokers	109788 (37.0)	10412 (35.0)	10187 (34.3)	< 0.001
		Previous smokers	8326 (28.0)	10209 (34.4)	12317 (41.4)	
		Current smokers	10405 (35.0)	9097 (30.6)	7215 (24.3)	
	PC2: Animal food consumers	Non smokers	10777 (36.3)	10825 (36.4)	9985 (33.6)	<0.001
		Previous smokers	10930 (36.8)	10422 (35.1)	9500 (32.0)	
		Current smokers	8012 (27.0)	8471 (28.5)	10234 (34.4)	
	PC3: Traditional Norwegian	Non smokers	9911 (33.3)	10648 (35.8)	11028 (37.1)	<0.001
		Previous smokers	9833 (33.1)	10389 (35.0)	10630 (35.8)	
		Current smokers	9975 (33.6)	8681 (29.2)	8061 (27.1)	
	PC4: Abstainers	Non smokers	6695 (22.5)	10790 (36.3)	14102 (47.5)	<0.001

		Previous smokers	9856 (33.2)	10640 (35.8)	10356 (34.8)	
		Current smokers	13168 (44.3)	8288 (27.9)	5261 (17.7)	

Supplementary Table 3. Principal component analysis: Main components loadings for four most prominent dietary patterns identified. All values, reported after varimax rotation.

		PC1: European	PC2: Animal food consumers	PC3: Traditional Norwegian	PC4: Abstainers
Beverages	Wine		0.135		-0.341
	Beer		0.206		-0.237
	Liquor	-0.141	0.206		-0.291
	Coffee	-0.184	0.1851		
	Tea	0.245			
Fat	Animal origin		0.163		
	Plant origin				0.220
	Other fat		0.205		0.101
Carbohydrates	Bread Wholemeal		0.108		0.331
	Bread White		0.164		
	Pasta and rice	0.298	0.109		
	Fruit	0.308	-0.116	0.157	
	Potatoes	-0.230	0.118	0.215	0.170
	Cereals	0.229	-0.12 32		
	Vegetable - root			0.350	0.103
	Vegetable - salad	0.359		0.100	-0.114
	Vegetable - other	0.290		0.194	
	Porridge				0.352
Eggs	Eggs		0.246		-0.147

Pizza	Pizza	0.163	0.255	-0.222	0.135
Fish and fish products	Cod liver	-0.166		0.364	
	Fish spread	0.144		0.288	
	Lean fish	-0.120		0.416	
	Oily fish	0.113		0.298	
	Fish products	0.114	0.152	0.261	0.110
Sugars and sugary food	Desserts and cakes				0.211
	Sweets	0.201	0.177	-0.164	0.151
	Jam				0.321
	Pancakes and waffles	0.208	0.128	-0.173	0.185
Meat and processed meat	Processed meat		0.378		0.112
	Red meat		0.351		
	Meat spread		0.235		
	Chicken	0.297	0.141		-0.130
	Stew and others	0.112	0.316		
Milk and dairy	High fat cheese	0.138			0.162
	Low fat cheese		-0.104	0.109	
	High fat dairy				
	Low fat dairy				0.128
	Yoghurt	0.211			
Cod liver oil	Cod liver oil			0.117	
